# Case Report: Postpartum hemorrhage associated with Dengue with warning signs in a term pregnancy and delivery

**DOI:** 10.12688/f1000research.7589.1

**Published:** 2015-12-21

**Authors:** Le Phi Hung, Tran Diem Nghi, Nguyen Hoang Anh, Mai Van Hieu, Nguyen Thien Luan, Nguyen Phuoc Long, Than Trong Thach

**Affiliations:** 1 University of Medicine and Pharmacy, Ho Chi Minh, Vietnam; 2 School of Medicine, Vietnam National University, Ho Chi Minh, Vietnam; 3 Hung Vuong Hospital, Ho Chi Minh, Vietnam

**Keywords:** Dengue with warning signs, pregnancy, uterine atony, postpartum hemorrhage, polyhydramnios

## Abstract

**Background:** Dengue infection during peripartum period, although rare in endemic regions, has challenged clinicians regarding its management, especially if a parturient woman experiences postpartum hemorrhage due to a classical risk factor of maternal bleeding.

**Case:** A full-term pregnant Vietnamese woman was diagnosed with polyhydramnios and Dengue with warning signs (DWS). She was administered platelet transfusion prior to delivery and then gave birth to a healthy newborn. After active management of the third stage of labor, the patient suffered a postpartum hemorrhage which was caused by uterine atony and accompanied with thrombocytopenia. Therefore, we decided to administer uterotonic drugs and additionally transfuse platelets.

**Conclusion:** We describe a case of postpartum hemorrhage caused by uterine atony and coinciding with Dengue infection during delivery period, which is a rare clinical entity. With timely detection and management, the patient was finally discharged without complications.

## Introduction

Dengue infection in parturient women living in endemic regions only accounts for 2.5% of all pregnancies ^1^. Besides the possibility of vertical transmission to newborns, Dengue infection may act as one of the potential risk factors of massive postpartum hemorrhage ^2^ as well as many other serious complications for mothers. Particularly, during the peripartum period, if the mother simultaneously experiences any bleeding conditions such as cesarean section, the hemorrhage might be exacerbated and life-threatening. Even though postpartum hemorrhage is one of the most aggressive obstetric complications, especially when it coexists with Dengue infection, there have been few papers in the medical literature reporting the management of such cases.

We hereby present a pregnant Vietnamese woman who neither respected the antenatal appointment schedule nor fulfilled prenatal tests. Prior to hospitalization in our obstetric hospital, she was diagnosed with Dengue with warning signs. The parturient woman, at the same time, suffered from polyhydramnios and uterine atony. After her delivery of a completely healthy infant, she suddenly had a severe postpartum hemorrhage. Owing to effective resuscitation, the patient and her neonate were finally discharged in stable condition. The one-week follow-up did not show any abnormalities.

## The case

A 34-year-old gravida III woman presented to our hospital at 39.5 weeks of gestation with no significant medical history. During her pregnancy period, she did not regularly follow the consultant schedule nor fulfill prenatal tests. At 39 weeks of gestation, the parturient woman was reported to have polyhydramnios by ultrasound and then admitted to a provincial hospital for observation. Unfortunately, we could not obtain the images confirming her polyhydramnios state.

On her fourth inpatient day in the provincial hospital, she suddenly had a continuous high-grade fever (39°C) associated with myalgia and arthralgia. The first complete blood count result showed a platelet count of 69.000/mm ^3^ and hematocrit of 38.8%. The patient was suspected to have Dengue and received symptomatic and supportive treatment with acetaminophen and intravenous fluids for 3 days. Vital signs and bleeding manifestations were also closely monitored. On the seventh inpatient day, the body temperature declined and then remained stable at 37°C. In the same day, the patient noted small petechiae sized 1–2 mm in diameter, around her arms and forearms. The platelet count at this time was 50.000/mm ^3^ and hematocrit was 40.8%. One day later, the patient abruptly experienced an intermittent hypogastric pain and had vaginal bleeding. She was transferred to our tertiary obstetric hospital where she was confirmed to have Dengue with warning signs.

On the day of admission, the patient was alert and the vital signs were unremarkable (blood pressure of 120/80 mmHg, temperature of 37°C, pulse of 100 beats/min and respiration rate of 20 breaths/min). On obstetric examination, the clinician recorded a 33 cm fundal height, fetal heart rate of 142 beats/min. The cervix was 2 cm dilated and 60% effaced. Regarding laboratory analysis, the rapid test for Dengue diagnosis revealed the following results: NS1 antigen (+), IgM Dengue (+) and IgG Dengue (-). Hematocrit increased to 45.2% and the platelet count showed a continuously downward trend to 32.000/mm ^3^. Liver and renal function tests as well as coagulation workup revealed no abnormalities. In the delivery room, the patient was administered 2 units of packed platelet concentrate in around 30 minutes before giving birth. The patient successfully delivered a healthy female infant weighing 3.500 grams by vaginal route with APGAR score of 8 and 9 after 1 and 5 minute(s), respectively. A uterine atony and a first-degree vaginal tear were recorded, leading to the loss of about 200 ml of blood. The third stage of labor was actively managed, then the perineal laceration was sutured and the retained products of conception were excluded. After that, the uterus was well contracted and a blood pressure of 120/72 mmHg and pulse of 86 beats/min were noted. The mother and newborn were transferred to the waiting room for observation. After 10 hours, the uterine contractions decreased suddenly and became boggy; the total amount of blood loss was about 1000 ml. The patient was diagnosed with postpartum hemorrhage due to uterine atony. Intravenous fluid resuscitation with 500 ml of 0.9% NaCl in two hours and a half was implemented. 20 IU of oxytocin diluted with 500 ml of Lactated Ringer’s solution was also administered intravenously with the infusion rate of 3 ml/min. In addition, the patient was given a dose of 200 mcg Methylergometrine by intramuscular injection. After 2 hours of intensive treatment, there was no further bleeding episodes and the normal range of vital signs were recorded. Repeated tests after childbirth showed moderate thrombocytopenia with a platelet count of 47000/mm ^3^ but no abnormal coagulation was detected. After bleeding was well-controlled with oxytocin, the clinicians decided to transfuse one more unit of platelets in order to increase the patient’s platelet concentration to a safe range. The platelet count and hematocrit were 52.900/mm ^3^ and 35.9% respectively, after transfusion. The patient was transferred to the department of postpartum care. The patient gradually recovered without any abnormality. The laboratory findings of the newborn were unremarkable, especially the negative serological test for detecting Dengue infection. Consequently, the patient and her neonate were discharged in a stable state after 7 inpatient days in our hospital. The one-week follow-up also did not show any abnormal conditions.

## Discussion

Several studies have suggested that Dengue infection can predispose full-term pregnant women to postpartum hemorrhage, even massive bleeding. Chotigeat *et al.* reported one case of a patient suffering from Dengue shock syndrome (DSS) which later developed postpartum hemorrhage ^4^. Thaithumyanon *et al.* described a of heavy bleeding following a C-section ^3^. The report of Chye JK. *et al.* described a parturient woman with substantial thrombocytopenia and abnormal coagulation which required intensive treatment ^5^. Our case report, however, may be the first detailed description of a severe postpartum hemorrhagic case involving uterine atony ^6^. Postpartum hemorrhage arose after vaginal delivery and was complicated by thrombocytopenia secondary to DWS. Diagnosis of DWS in late pregnancy requires a high degree of clinical suspicion because signs and symptoms frequently seen in Dengue may be confused with preeclampsia complicated by HELLP syndrome ^5, 7^. Moreover, physiological hemodilution in late pregnancy ^8^ may obscure the degree of vascular leakage and challenge clinicians to evaluate it as well as to treat the patient appropriately. In our case, the patient experienced high fever, marked thrombocytopenia, hematocrit above the upper limit of the normal value and particularly, no clinical or laboratory findings suggesting preeclampsia with HELLP syndrome; this made the diagnosis of Dengue more probable. The diagnosis was confirmed by positive results from NS1 antigen and IgM/IgG rapid tests. We chose the rapid diagnosis test due to the fact that our laboratory’s resources were limited and the patient needed emergency management.

Vaginal delivery may be allowed without prophylactic platelet transfusion even though platelet count falls below 50000/mm ^3^ ^6^. Concern about the risk of profound bleeding due to uterine atony, however, led to our decision to raise the platelet count. Optimal timing for transfusion remains controversial, but it should not be too far away from the time of delivery ^9^, since a study demonstrated that in DSS patients, platelet count declined quickly and returned to pre-transfusion level only in several hours ^10^. In our case, platelet transfusion was initially performed 30 minutes before giving birth and once again 8 hours after delivery. Repeated post-transfusion blood analyses, however, indicated significant decrement of platelet count, which was in the same pattern with the findings of the study mentioned above ^10^. Regardless of prophylactic platelet transfusion, according to a previous retrospective study, clinical bleeding in an adult patient with Dengue infection may still occur ^11^, partially due to the intricate affect of Dengue infection on the hemostatic system ^12^. Therefore, in such cases, platelet transfusion might not be overemphasized and management of other risk factors of bleeding in the obstetric background, such as polyhydramnios in this case, must also be considered. In spite of successful active management of the third stage of labor, the lack of close follow-up thereafter, which was partly due to hospital overload, made us unable to detect earlier delayed postpartum hemorrhage. Recurrence of uterine atony might be the major cause owing to the finding of boggy, hypotonic uterus. The fact that uterotonic drugs controlled bleeding effectively before platelet transfusion was performed also indicated the diagnosis of uterine atony. Treatment with uterotonic drugs was essential because myometrial contraction acts as the major factor preventing blood loss after natural delivery ^13^, even if there are abnormalities in the hemostatic system.

Medical literature recorded many cases of vertical transmission of Dengue virus ^3– 5^. Dengue infection in mothers at or near the time of delivery may predispose neonates to suffer from many dangerous complications because the vast majority of antibodies which are transferred across placenta do not have a protective effect ^14^ but actually enhance the entry of virus into the host cells ^15^. As the result of this, careful neonatal follow-up in the hospital must be performed. Time to observation remains elusive, but several studies have reported that time of febrile onset varied between 1 and 11 days of life ^16^. In our case, a 14 day observation was not feasible given the background of hospital overload. As a result, we kept the baby in the hospital during the first 7 days of age, and then discharged mother and child after counselling the family to visit for a check-up one week later.

## Conclusion

A case of postpartum hemorrhage resulting from uterine atony and accompanied with Dengue infection in term pregnancy and delivery has rarely been reported in obstetric medical literature. Until now, no standard treatment has been proposed. Our current case, fortunately, reported successful management with conservative treatment and timely uterotonic administration.

## Consent

Written informed consent for publication of their clinical details was obtained from the patient.
