# Supplementary material for: Case Report: Postpartum hemorrhage associated with Dengue with warning signs in a term pregnancy and delivery
Source: F1000Res. 2015 Dec 21;4:1483. [Version 1] doi: 10.12688/f1000research.7589.1 (PMC4712770; doi:10.12688/f1000research.7589.1)
Supplement: Peer review history

# Peer review: Referee response for version 1

**DOI:** 10.5256/f1000research.8172.r11878

**Published:** 2016-01-11

**Authors:** Gamal Serour

**Affiliations:** 1 Department of Obstetrics and Gynaecology, Al Azhar University, Cairo, Egypt

Thank you very much for asking me to referee this interesting paper. It is a good paper which will be interesting to the readers. I recommend its acceptance for indexation

I have read this submission. I believe that I have an appropriate level of expertise to confirm that it is of an acceptable scientific standard.

# Peer review: Referee response for version 1

**DOI:** 10.5256/f1000research.8172.r11676

**Published:** 2016-01-04

**Authors:** Michael Paech

**Affiliations:** 1 School of Medicine and Pharmacology, University of Western Australia, Perth, Australia

This was an interesting case report for those not familiar with clinical cases of Dengue fever, although postpartum hemorrhage is not unexpected in a woman with multiple risk factors including multiparity, polyhydramnios, absent antenatal care and Dengue-induced thrombocytopenia. 

The report is well written apart from a few English phrases that are not quite correct (check first couple of sentences of the Discussion for example). I think it would be helpful to mention other diagnostic features (neutropenia, bradycardia, positive tourniquet test and full laboratory assessment). Perhaps also some more detail about the time course /  natural history of Dengue with respect to pathologies. The decision to use a precious resource such as platelets after postpartum hemorrhage had resolved with oxytocic therapy may not have been appropriate and a caveat should be included.

I have read this submission. I believe that I have an appropriate level of expertise to confirm that it is of an acceptable scientific standard.
